# Systemic deletion of Atp7b modifies the hepatocytes’ response to copper overload in the mouse models of Wilson disease

**DOI:** 10.1038/s41598-021-84894-3

**Published:** 2021-03-11

**Authors:** Abigael Muchenditsi, C. Conover Talbot, Aline Gottlieb, Haojun Yang, Byunghak Kang, Tatiana Boronina, Robert Cole, Li Wang, Som Dev, James P. Hamilton, Svetlana Lutsenko

**Affiliations:** 1 Department of Physiology, Johns Hopkins Medical Institutes, 725 N Wolfe street, Baltimore, MD 21205 USA; 2 Core Analysis Unit, Johns Hopkins Medical Institutes, Baltimore, MD 21205 USA; 3 Department of Molecular and Comparative Pathobiology, Johns Hopkins Medical Institutes, Baltimore, MD 21205 USA; 4 Mass Spectrometry and Proteomics Facility, Johns Hopkins Medical Institutes, Baltimore, MD 21205 USA; 5 Department of Medicine, Johns Hopkins Medical Institutes, Baltimore, MD 21205 USA; 6 Present Address: Novartis Institutes for Biomedical Research, Cambridge, MA USA

**Keywords:** Diseases, Pathogenesis

## Abstract

Wilson disease (WD) is caused by inactivation of the copper transporter Atp7b and copper overload in tissues. Mice with Atp7b deleted either globally (systemic inactivation) or only in hepatocyte recapitulate various aspects of human disease. However, their phenotypes vary, and neither the common response to copper overload nor factors contributing to variability are well defined. Using metabolic, histologic, and proteome analyses in three *Atp7b*-deficient mouse strains, we show that global inactivation of *Atp7b* enhances and specifically modifies the hepatocyte response to Cu overload. The loss of *Atp7b* only in hepatocytes dysregulates lipid and nucleic acid metabolisms and increases the abundance of respiratory chain components and redox balancing enzymes. In global knockouts, independently of their background, the metabolism of lipid, nucleic acid, and amino acids is inhibited, respiratory chain components are down-regulated, inflammatory response and regulation of chromosomal replication are enhanced. Decrease in glucokinase and lathosterol oxidase and elevation of mucin-13 and S100A10 are observed in all Atp7b mutant strains and reflect the extent of liver injury. The magnitude of proteomic changes in *Atp7b*^*−*/*−*^ animals inversely correlates with the metallothioneins levels rather than liver Cu content. These findings facilitate identification of WD-specific metabolic and proteomic changes for diagnostic and treatment.

## Introduction

Wilson disease (WD) is an autosomal-recessive disorder caused by inactivating mutations in the copper (Cu) transporter ATP7B and subsequent accumulation of Cu in tissues, especially in the liver^1^. Although WD is a monogenic disorder, the time of disease onset and specific presentations vary significantly^2,3^. This variability reflects the existence of the phenotype-modifying factors^4–7^ and greatly complicates the diagnosis, treatment, and the mechanistic understanding of WD^8,9^.

Animal models have been extensively used to investigate WD pathogenesis. These studies identified inhibition of nuclear receptors^10–12^, epigenetic modifications^13,14^, and mitochondria dysfunction^15,16^ as important hallmarks of the disease. Despite many insightful findings, the consistent picture of metabolic changes associated with Atp7b inactivation in the liver (the “WD signature”) has been slow to emerge. The observed differences between the phenotypes of Atp7b-deficient animals, as well as human WD, led to uncertainties about how to compare and best utilize the available animal models. Specifically, the genetically engineered mice with global (in all tissues) inactivation of Atp7b (*Atp7b*^*-/–*^ mice) recapitulate many classic features of human WD^17^. These mice rapidly accumulate Cu in the liver, have impaired Cu incorporation into ceruloplasmin, and show clear signs of liver inflammation and dysfunction^12,18^. The inbred mice with mis-sense mutations in *Atp7b* (*tx* and *txj* mice) also accumulate Cu in the liver and show decreased mitochondrial function, but the disease progression in these animals is slower and the phenotype is milder compared to *Atp7b*^*−*/*−*^ mice^19^. In inbred LPP rats, the loss of Atp7b expression is associated with marked mitochondria impairment and death from liver failure^20^. Lastly, mice with Atp7b deleted only in hepatocytes (*Atp7b*^*ΔHep*^) accumulate Cu in the liver, lack active ceruloplasmin, but do not show hepatocyte ballooning or inflammatory response. Instead, these animals have mild obesity and develop liver steatosis at old age^21^.

It has been unclear whether these phenotypic differences reflect genetic variability between the background strains, species differences, variation of animal ages at which experiments were done^21,22^ or specific research focus of a given study. Consequently, we asked what are the major similarities between the different mouse models of WD and what factors may influence their liver phenotype. To answer these questions, we systematically compared three different age-matched mouse strains with Atp7b inactivated either globally or only in hepatocytes. Comparison of liver morphology, function, and proteomes identified the pathways and molecules that were affected in all Atp7b-deficient animals and highlighted a significant impact of systemic Atp7b inactivation on liver morphology and function.

## Materials and methods

### Animal husbandry

All experimental procedures for using mice in this study were approved by the Johns Hopkins University Animal Care and Use Committee (JHU ACUC, protocol # MO15M27) and performed accordingly. *Atp7b*^*−*/*−*^ mice were maintained either on a C57BL/6 × 129S6/SvEv background (*Atp7b*^*−*/*−*^ hybrid) or were backcrossed with C57BL/6 J mice 10 times and then maintained on a C57BL/6 J background (*Atp7b*^*-/–*^ B6). The study design, execution, and reporting followed the ARRIVE guidelines. Each litter was kept in a separate cage; different cages were located in the same room, on the same rack. To decrease confounding effects, animals from different litters were used. Whenever possible, the same number of animals per each group was used, the typical group size was 6 animals. Previous studies determined that this group size provides a reliable and statistically significant comparison of key phenotypic parameters, such as hepatic copper content and histomorphology at 20 weeks. When available, up to 11 animals per group were included. In some experiments, fewer (4–5) animals for of *Atp7b*^*−*/*−*^ hybrid strain were included, because this strain has been previously described in detail and fewer animals were needed to replicate the phenotype; specific number of animals for each experiment is indicated in the figure legends. Animals used for each experiment were selected randomly and no animals of correct genotype was excluded in final analysis.

**Figure 1:**
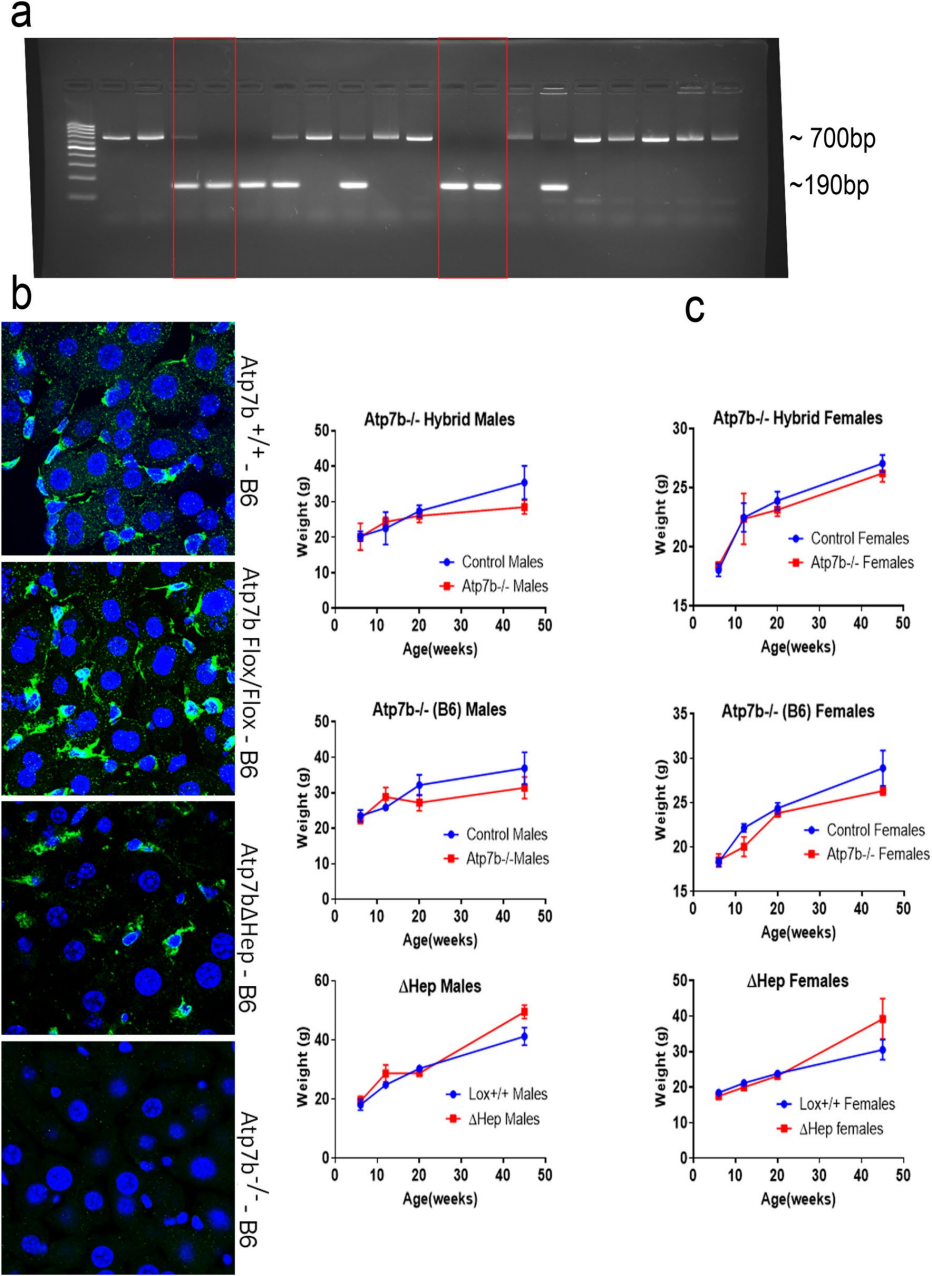
Characteristics of *Atp7b*^*−*/*−*^ mice on C57BL/6 J background (*Atp7b*^*-/–*^*B6*). (**a**) Genotyping littermates to identify the Cre-mediated deletion. The 190 bp product is indicative of the desired deletion within *Atp7b* (red boxes). The 700 bp product reflects the wild-type genotype. (**b**) The livers from 6-weeks old mice were sectioned, immuno-stained with anti-ATP7B antibody (green) and DAPI (nuclei, blue), and then imaged using an LSM 800 confocal microscope. In the wild-type (*Atp7b*^+*/*+^) and *Atp7b*^*Flox/Flox*^ livers, ATP7B (green) is detected in both hepatocytes (large cells with large nuclei) and non-parenchymal cells (small cells with small nuclei). *Atp7b*^*ΔHep*^-B6 mice show staining predominantly in small non-parenchymal cells. No ATP7B staining is seen in *Atp7b*^*-/–*^*B6* livers*.* (**c**) Comparison of the weight gain curves for Atp7b-mutant mice (blue), males and females, and the background-matched control animals (red); each point represents an average of data from 3–11animals. Individual weights and statistical analysis of weight differences between the *Atp7b*^*−*/*−*^ mice and the background-matched wt controls at 20 weeks and at 45 weeks are given in Supl. Fig. S1.

For genotyping, DNA from tail clips of littermate animals was extracted using an Accustart II Mouse genotyping Kit, the PCR was performed using primers described in the Suppl. Table 1, and the products were analyzed on agarose gel (Fig. 1a). The *Atp7b*^*-/–*^*B6* mice, *Atp7b*^*−*/*−*^ hybrid mice, and a previously generated *Atp7b*^*ΔHep*^*-B6* strain along with the control mice on the same respective genetic background and *Atp7b*^*Lox/Lox*^-B6 controls, were fed normal chow and housed following the JHU ACUC guidelines. Reaching certain age defined the endpoint of the experiment; there were two main end-points – 20 weeks and 45 weeks after birth. Animal weight was monitored from 6 to 45 weeks. The control age- and sex-matched animals were either wild type or heterozygous on the respective genetic background. The lack of phenotype and normal liver histology and function in heterozygous mice allowed us to use these animals as controls for phenotypic analyses. For proteomic studies, only WT animals (both the hybrid and B6 background) were used as controls.

### Statistical analysis

Statistical analysis was performed using Prism version 8.0 (GraphPad Software) for all experiment except mass-spectrometry studies (see below). One-way analysis of variance (ANOVA) or a two-tailed t-test was used for the data analysis, as indicated in figure legends. All data were included in the analyses; the results are given as the mean ± standard deviation (SD). A *p* value of < 0.05 was considered significant.

### Tissue and serum collection

Mice were sacrificed at the same time of the light cycle by isoflurane inhalation, and tissue and serum were collected at 7, 12, 20, 31, and 45 weeks after birth. Blood was collected following cardiac puncture, placed into BD Microtainer tubes, allowed to clot for 30 min at room temperature (RT) and then centrifuged at 2,000 × g for 10 min at 4ºC. The serum was collected and stored at −80 °C until further analysis. Measurements of serum metabolites were done at the Johns Hopkins University Phenotyping and Pathology Core.

### Histology

Liver tissues were collected and a piece of each liver was placed in 10% formalin for subsequent sectioning, Hematoxylin and Eosin (H&E) staining. Sections were also stained for collagen for 1 h using Picro-Sirius red solution (0.1% Direct Red 80 (Sigma #365,548) in 1.3% saturated picric acid (Sigma #P6744) after 8 min hematoxylin nuclei stain. Slides were washed with 0.5% acetic acid twice before dehydration and mounting. Images were acquired using an Olympus light color microscope and the Sirius red positive areas were measured using image J. The rest of the liver was kept at −80 °C for further analysis.

### Copper measurements

Liver pieces (50–100 mg) were weighed and digested in 200 µl concentrated nitric acid (_~_70%) at 70 °C for 4 h. After digestion, the samples were cooled to RT and 300 µl of HPLC grade water was added. The samples were centrifuged at 2,000 g for 5 min, and the supernatant was transferred to a 1.7 ml microtubes, diluted with HPLC grade water to a final nitric acid concentration of < 2%. Cu measurements were done using a PerkinElmer Atomic Absorption Spectrometer PinAAcle 900 T. For each biological replicate, three technical replicates were used. The Cu levels were normalized to tissue weight and reported as µg/mg wet tissue.

### Triglyceride measurements

Liver tissue lysates were prepared by homogenizing 50–100 mg of tissue in 50 mM HEPES (1/1000 Igepal 150 mM NaCl**,** 0.25 M sucrose**,** 0.5uM AEBSF**,** ¼ tablet protease inhibitor EDTA Free) using the hand-held battery-operated homogenizer VWR pellet mixer on ice. The homogenates were centrifuged at 700 × g for 15 min to remove debris. The supernatant was collected and protein concentration was estimated by BCA assay. Triglycerides were measured by analyzing lysates containing 10 µg of total protein using Triglyceride assay (ThermoFisher Scientific) and Pointe scientific triglyceride standard for calibration curve. The values were normalized to protein concentration and reported as ng/µg protein.

### Quantitative polymerase chain reaction

Fifty to one hundred milligrams of liver tissue were homogenized using a battery-operated homogenizer. RNA was isolated using Trizol according to the manufacturer’s protocol (Ambion Life Technologies) and quantified using an IMPLEN Nano photometer. The quality of RNA was determined using the A260/280 ratio and samples with values above 1.8 were used for further analysis. Gene expression was analyzed using a Power SYBR Green RNA-to Ct 1-step kit (Applied Biosystems); 50 ng of RNA was used in the analysis. Ct values were normalized to the GAPDH mRNA used as an endogenous control. Relative gene expression was determined by comparing all samples to one wild type C57BL/6 × 129S6/SvEv liver.

### ATP7B staining in the liver

Pieces of liver tissue were embedded in optimal cutting temperature (OCT) medium and kept at −80 °C until sectioning. Tissue blocks were cut into 10 µm sections and mounted onto Fisherbrand superfrost plus microscope slides. The sections were kept at −80 °C until analysis. OCT was removed by washing 2 × 5 min in phosphate buffered saline (PBS). Antigen retrieval was achieved by incubating sections in 1:10 Histo VT One solution at 65ºC for 40 min. The sections were blocked in 1% bovine serum albumin (BSA) and 0.1% Tween in 1 × PBS (PBST). After blocking, the sections were incubated in 1:200 rabbit anti-Atp7b antibody (EPR6794, Abcam) overnight at 4ºC. Sections were washed in PBST 4 × 5 min each. Secondary incubation was done in 1:500 donkey anti-rabbit 488 green antibody for 1 h. The sections were then washed twice with PBST and twice with PBS for 5 min each. Staining of nuclei was done by incubating slides for 5 min in 1:1000 Hoeschst 3342, trihydrochloride (Invitrogen) and then washing in PBS for 5 min. Sections were mounted using ProLong Gold antifade reagent and imaged with Zeiss LSM 800 microscope.

### TMT labeling and mass-spectrometry

For comparative analysis of proteomes presented in this study, two different protein sets were used. One contained 3 biological replicates (different animals) for each genotype: WT-B6, *Atp7b*^*−*/*−*^-B6, and *Atp7b*^*ΔHep*^-B6 animals. This dataset was used to determine and compare the effects of global and hepatocyte specific inactivation for animals on the same genetic background. The second dataset contained two WT-hybrid, two WT-B6, one *Atp7b*^*−*/*−*^-B6, and four *Atp7b*^*−*/*−*^ hybrid liver samples (all biological replicates). This dataset was used for two purposes. First, to identify proteins and pathways changed in the *Atp7b*^*-/–*^hybrid livers. Second, to verify that the changed pathways analyzed in different mass-spectrometry runs (set 1 and set 2) can be compared even when the identified proteins in these sets overlapped only partially. This was done by comparing the changed cellular processes/pathways for WT-B6 versus *Atp7b*^*−*/*−*^-B6 pairs in the first and second database.

Proteins (100 ug) were first reduced with 3 mL of 15 mg/mL dithiothreitol (DTT) in 100 mM triethyl ammonium bicarbonate (TEAB) for 1 h at 57ºC with mixing. The pH was adjusted to 8.0 with 10 µL of 500 mM TEAB and proteins alkylated using 3.6 µL of 36 mg/mL iodoacetomide for 10–15 min at RT in the dark. 8 × TCA/acetone volume was added on ice and proteins precipitated at −20 °C overnight. Protein pellets were washed with iced acetone, dried, re-constituted in 750 µL 9 M urea, sonicated and loaded on 30 kDa filters (Millipore) to remove sucrose. The proteins on 30 kDa filter were proteolyzed with 6 µg Trypsin (Pierce) in 350 µL 25 mM TEAB at 37ºC overnight. The peptides eluted and dried.

Peptides were then reconstituted in 100 µL of 100 mM TEAB buffer and labeled with TMT 10 plex reagent (Thermo Fisher-Pierce) according to manufacturer’s protocol. The TMT-labeled peptides (250 mg) were dried and then re-constituted in 2 mL bRP buffer A (10 mM TEAB in water) and injected over 8 min at 250ul/min. Peptides were fractionated by basic reverse chromatography using 85 min gradient from 100% solvent A (10 mM TEAB in water) to 100% solvent B (90% acetonitrile/10 mM TEAB) at 250 ml/min on a XBridge C18 Column (5 µm, 2.1 × 100 mm column, Waters) with a XBridge C18 Guard Column, 5 µm, 2.1 × 10 mm (Waters) on Agilent HPLC system 1200 series. Eighty-four fractions were collected and re-combined into 24 fractions (10.42 µg per fraction) for LC–MS/MS analysis.

#### LC–MS/MS analysis

Standard protocol^43^ has been used to analyze the fractionated peptides. Specifically, peptides in each fraction were dissolved in 125 µL of the loading buffer (2% acetonitrile/0.1% formic acid) and 4 µL aliquots were analyzed on a 75 µm × 150 mm ProntoSIL-120–5-C18 H column 3 µm, 120 Å (BISCHOFF) using linear gradient (2%–90% acetonitrile/0.1% formic acid gradient over 100 min at 300 nl/min). Eluted peptides were then sprayed into an Orbitrap-Lumos-Fusion mass spectrometer at 2.4 kV, survey scans were acquired within 375–1600 Da m/z with a dynamic exclusion of 15 s. Precursor ions were fragmented and both precursor ions and fragments were analyzed at a resolution of 120,000 and 60,000, respectively.

Tandem MS2 spectra (signal/noise > 2) were processed by Proteome Discoverer (v2.3 ThermoFisher Scientific) using 1Node, PD2.3 and recalibration with appropriate database. MS/MS spectra were searched with Mascot v.2.6.2 (Matrix Science, London, UK) against RefSeq2017_83_mus_musculus database and then processed within the Proteome Discoverer and Percolatorto to identify peptides with a confidence threshold of 1% False Discovery Rate. Only Peptide Rank 1 were considered.

#### Statistical treatment of data

The Proteome Discoverer data were imported to Partek Genomics Suite 7.0 (Partek Inc. Saint Louis MO) and processed using the standard protocol^44^. Discoverer assigns the detected mass spectra to an NCBI RefSeq identifier; for each identifier the median of the multiple spectra values was calculated to produce a single value of the corresponding protein abundance. The spectra with a unique, single, RefSeq and a Proteome Discoverer Isolation Interference value of < 30% were used for further evaluation of the TMT runs. The values of each data set's samples were quantile normalized.

To determine the relative protein expression levels of the different biological classes in Partek, their biological replicates’ quantile normalized log2 values underwent two-tailed one-way ANOVA t-test analysis wherein the proteins’ relative expression levels were reported as fold change and the statistical significance of that change reported as *p* value. Different pairs of biological class were compared with each protein/gene yielding linear and log2 fold change values. Principal component analyses used correlation of all the proteins’ expression levels to group the various samples’ similarity to each other.

To examine and graphically depict differential protein expression, these analyses’ results were imported into Spotfire DecisionSite with Functional Genomics v9.1.2 (TIBCO Spotfire, Boston, MA, USA). To characterize these fold changes, their log2 values (the conventional “log ratios” or “log2-fold changes that demonstrate a normal distribution) underwent standard deviation, SD, analyses allowing us to set appropriate fold change thresholds of differential expression. Volcano plots, which present both fold change and *p* value, on the X- and Y-axes respectively, were created to provide a unified view of the protein expression comparisons between cell classes.

#### Analysis of metabolic and signaling pathways

A functional annotation analysis of changes in liver proteins was done using Ingenuity software package (*IPA, Thermo Fisher Scientific).* The Partek-processed sets of proteins with changes in their intensity exceeding two standard deviations (2 s) of means were imported into IPA and subjected to Ingenuity Core analysis. This analysis was done individually for *Atp7b*^*−/–*^ hybrid proteome (in comparison to WT-hybrid), *Atp7b*^*-/–*^*B6* proteome in comparison to WT-B6, and *Atp7b*^*ΔHep*^-B6 proteome (in comparison to WT-B6) to identify and visualize the most significantly altered canonical pathways, and molecular and cellular processes. The comparison option was used to identify proteins commonly changed in all Atp7b-mutant livers.

## Results

### The phenotype of Atp7b^-/–^B6 mice is similar to Atp7b^-/–^hybrid mice and differs from the Atp7b^ΔHep^-B6 phenotype

Previously, we observed a very different liver histopathology for the global Atp7b knockout mice and mice with Atp7b deleted selectively in hepatocytes, despite similar Cu elevation in their livers^21^. One potential reason for this dissimilarity could be difference in genetic backgrounds of these animals. Original *Atp7b*^*−*/*−*^ mice were generated on a hybrid C57BL/6 × 129S6/SvEv background (*Atp7b*^*-/–*^hybrid), whereas *Atp7b*^*∆Hep*^ mice were produced on C57BL6 background (*Atp7b*^*∆Hep*^-B6). Genetic backgrounds may significantly influence animals’ metabolism and modify the course of a disease^23^. To clarify the role of a genetic background in the WD phenotype in mice, we transferred the *Atp7b* deletion from a hybrid background to C57BL6 background; we then compared *Atp7b*^*-/–*^B6 mice to *Atp7b*^*-/–*^hybrid mice of the same age. We also examined how additional loss of Atp7b in extrahepatic tissues affects liver phenotype—by comparing the global (*Atp7b*^*−*/*−*^-B6) and the hepatocyte-specific (*Atp7b*^*ΔHep*^*-B6*) knockouts on the same genetic background.

The deletion of Atp7b in *Atp7b*^*−*/*−*^-B6 mice was confirmed by PCR (Fig. 1a) and immunostaining of Atp7b in liver sections. (Fig. 1b). Weight changes in *Atp7b*^*−*/*−*^ B6 mice, measured from 7 to 45 weeks after birth showed that *Atp7b*^*−*/*−*^-B6 females weighed similarly to the age-matched WT-B6 animals; whereas *Atp7b*^*-/–*^B6 males had a significantly lower body weight compared to WT-B6 controls (Fig. 1c and Fig. S1). These weight patterns resembled those of *Atp7b*^*-/–*^hybrid animals (Fig. 1c and Fig. S1), whereas the lower weight of *Atp7b*^*-/–*^B6 mice was in a stark contrast to an excessive weight of *Atp7b*^*ΔHep*^*-B6* mice, especially males (Fig. 1c and^21^). Taken together, the weight patterns suggested that global inactivation of Atp7b impacts the metabolic status of animals differently than the hepatocyte-specific deletion of Atp7b.

**Figure 2:**
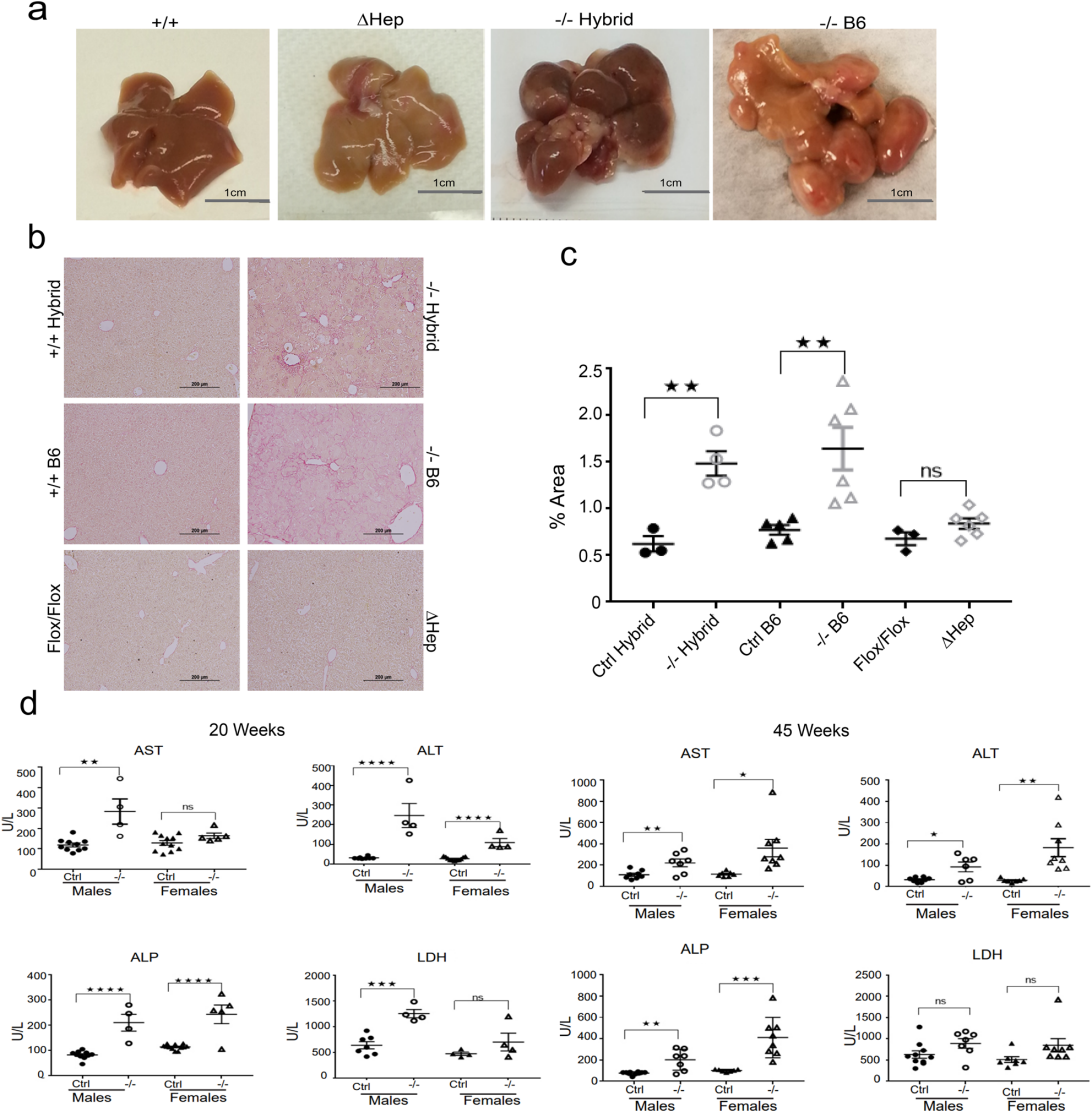
Liver morphology and function in Atp7b mutant animals. (**a**) Representative images of a whole liver from the wild-type mice (+/+), mice with Atp7b inactivated only in hepatocytes (Δ*Hep*) and the mice with Atp7b inactivated globally on two different background strains (^*−*/*−*^ Hybrid and ^−/–^B6) at 45 weeks. (b) Sirius Red staining illustrate the presence of fibrosis in mice with global inactivation of Atp7b (*−*/*−* Hybrid and *−*/*−* B6) but not in the hepatocyte specific knockout (*ΔHep*) or background-matched controls at 20 weeks. The images were collected at 10X magnification using an Olympus color station; representative images are shown; n = 3–6 per each genotype (c) Quantitation of Sirius Red staining intensity, n = 3–6**.** The unpaired two tailed t-test was used to evaluate significant changes between the pairs of mutants and respective controls. **p value ≤ 0.01, *p* > 0.05 is not statistically significant, (ns). (**d**) Serum liver enzymes in *Atp7b*^*−*/*−*^ B6 mice at 20 weeks and 45 weeks (n = 4–10). One-way ANOVA was used to identify significant changes between groups. *p value < 0.05, **p value < 0.01, ***p value ≤ 0.001 and ****p value ≤ .0001. *p* value > 0.05 is not statistically significant, (ns). “Ctrl”—control group—included wild-type and heterozygous animals on the corresponding genetic background.

Analysis of gross liver morphology at 45 weeks supported this conclusion. The *Atp7b*^*-/–*^*B6* livers showed a highly abnormal overall morphology, characterized by disruption and replacement of normal lobe structures with multiple hyperplastic nodules (Fig. 2a). These changes, again, resembled the *Atp7b*^*-/–*^hybrid phenotype and were not observed in the age-matched *Atp7b*^*ΔHep*^*-B6* mice (Fig. 2a and^21^).

The Sirius Red staining revealed presence of fibrosis in both global Atp7b knockouts, but not in *Atp7b*^*ΔHep*^*-B6* livers (Fig. 2b,c). To further evaluate liver function, we measured levels of transaminases in the serum. At 20 weeks, *Atp7b*^*-/–*^B6 mice showed significantly elevated AST, ALT, ALP when compared to WT-B6 mice, especially in males (Fig. 2d). The LDH levels were significantly decreased only in males. At 45 weeks, both sexes of *Atp7b*^*-/–*^B6 mice showed significant increases in the AST, ALP, and ALT values, in agreement with similar gross morphological changes of their livers.

### Metabolic consequences of hepatocyte-specific and global inactivation of Atp7b are different

**Figure 3:**
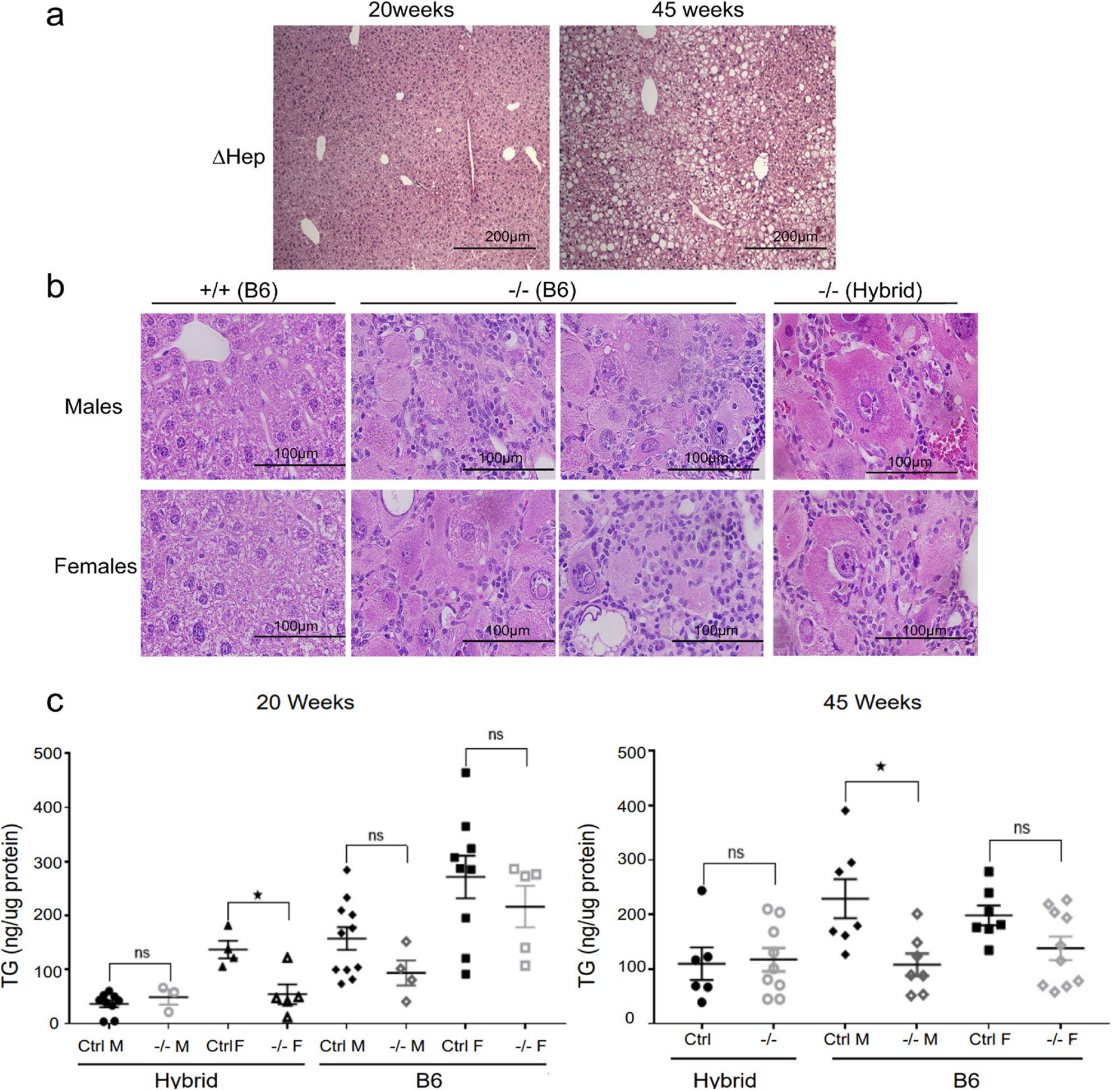
Global and hepatocyte-specific deletion of Atp7b have different impact on lipid metabolism. (**a**) The representative H&E staining of liver section illustrates the development of steatosis in *Atp7b*^*ΔHep*^-B6 animals (ΔHep), in agreement with the previous reports (^21^); (**b**) the lack of liver steatosis in *Atp7b*^*−*/*−*^-B6 and *Atp7b*^*-/–*^hybrid mice (males and females). (**c**) Triglyceride concentrations in the livers of *Atp7b−*/*−* hybrid (Hybrid) and *Atp7b*^*-/–*^B6 (B6) mice. The one-way ANOVA was used for statistical analysis *p value ≤ 0.05 was considered statistically significant, *p* > 0.05 is not statistically significant, (ns). “Ctrl”—control group—included wild-type and heterozygous animals on the corresponding genetic background.

We further analyzed the metabolic status of *Atp7b*^*-/–*^*-B6* livers using histologic stains and triglyceride measurements. Unlike *Atp7b*^*ΔHep*^*B6* animals (but similarly to *Atp7b*^*−*/*−*^ hybrid mice), *Atp7b*^*−*/*−*^*B6* mice did not accumulate lipid droplets in the cytoplasm of hepatocytes (^21^ and Fig. 3a,b). Instead, *Atp7b*^*-/–*^B6 mice showed progressive, chronic mononuclear hepatitis, hepatocyte loss, and degeneration (Fig. 3b). In this regard, *Atp7b*^*−*/*−*^-B6 mice were again similar to *Atp7b*^*−*/*−*^ hybrid animals, which had a pronounced inflammatory response and no significant steatosis (Fig. 3b). The lack of hepatic fat accumulation was confirmed by direct measurements of hepatic triglycerides (Fig. 3c). Neither *Atp7b*^*-/–*^B6 nor *Atp7b*^*−*/*−*^ hybrid livers had elevated triglycerides (Fig. 3c). *Atp7b*^*-/–*^B6 males showed a significant decrease of liver triglycerides at 45 weeks; *Atp7b*^*-/–*^hybrid females also had lower mean values, but variability of data was high, and the difference was not statistically significant.

**Figure 4:**
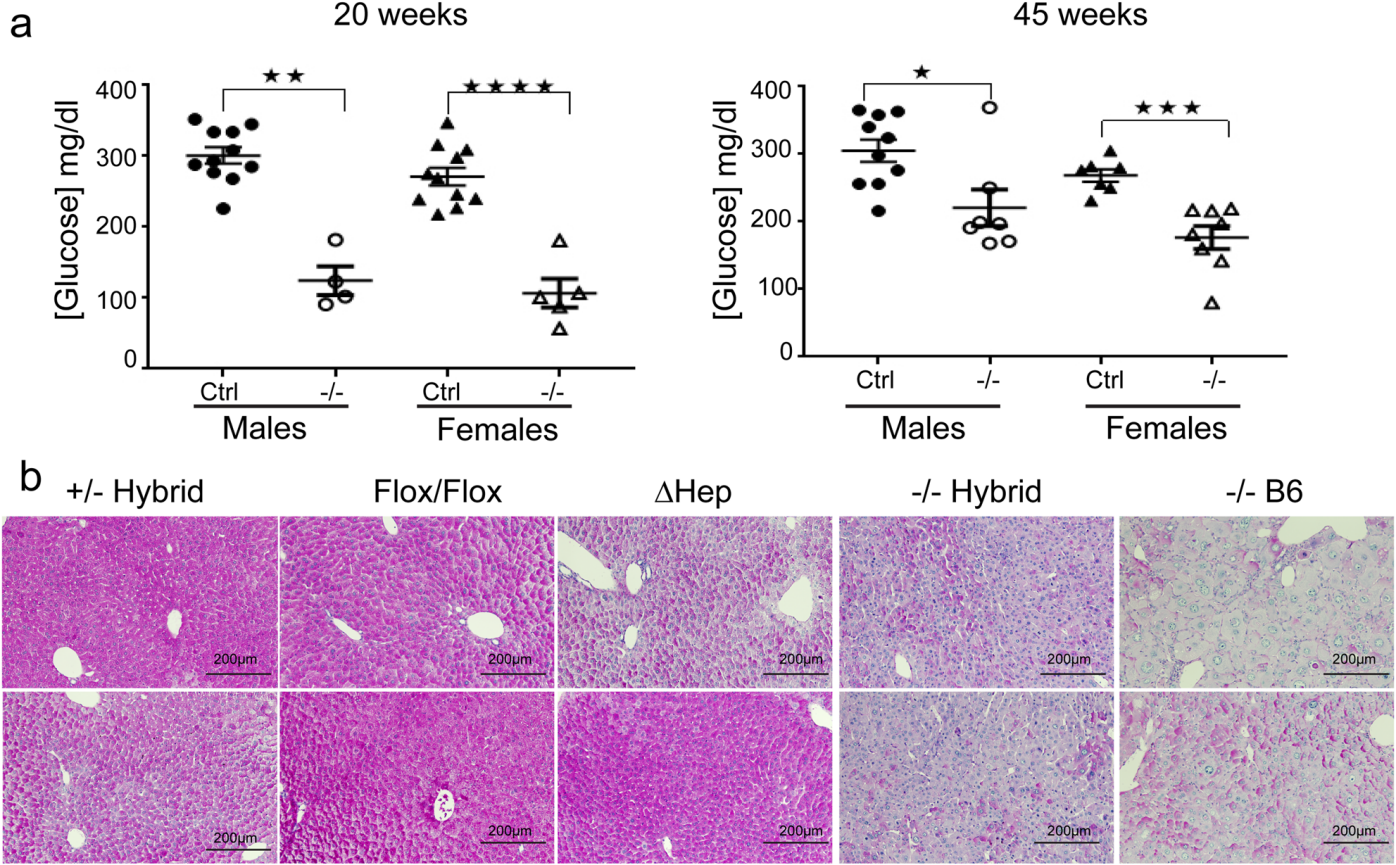
Atp7b global knockouts show similar changes in glucose and glycogen levels. (**a**) Glucose concentration in the serum of non-fasted *Atp7b*^*−*/*−*^-B6 male and female mice. Serum glucose was measured from animals following sacrifice. The difference between each pair of mutant and control animals was analyzed using unpaired two-tailed T test. The *p* value ≤ 0.05 was considered statistically significant and *p* > 0.05 not statistically significant, (ns); *p value ≤ 0.05, **p ≤ 0.01, ***p ≤ 0.001, and ****p ≤ 0.0001. (**b**) Representative PAS staining of control (*Atp7b*^+*/-*^ Hybrid), *Atp7b*^*Flox/Flox*^-B6 (Flox/Flox) *Atp7b*^*ΔHep*^-B6 (ΔHep), *Atp7b*^*-/–*^hybrid (*−*/*−* Hybrid), and *Atp7b*^*-/–*^B6 (*−*/*−* B6) at 20 weeks. PAS staining was performed on 10 µm paraffin embedded tissue sections following formalin fixation. The images were collected at 10X magnification using an Olympus color station. “Ctrl”—control group—included wild-type and heterozygous animals on the corresponding genetic background. N = 4–8 per group; two different animals per each genotype are shown.

We also observed similar changes in a serum glucose in global knockouts, and differences between *Atp7b*^*-/–*^B6 and *Atp7b*^*ΔHep*^-*B6* mice with respect to their glucose status. In *Atp7b*^*-/–*^*B6* mice, the levels of serum glucose were lower compared to control-B6 littermates at both 20 and 45 weeks (Fig. 4a), whereas serum glucose was elevated in *Atp7b*^*ΔHep*^-*B6* mice, as we reported previously (^21^, data are not shown to avoid duplication with the published data). Different status of glucose metabolism between the hepatocyte-specific and global knockouts was associated with differences in glycogen storage. PAS staining demonstrated glycogen depletion in both global knockouts and no significant glycogen depletion in *Atp7b*^*ΔHep*^*B6* livers (Fig. 4b).

Taken together, these results strongly suggest that independently of the strain genetic background the hepatocyte-specific and global inactivation of ATP7B have different consequences for liver morphology and metabolism.

### Genetic background influences the timeline of pathology onset

**Figure 5:**
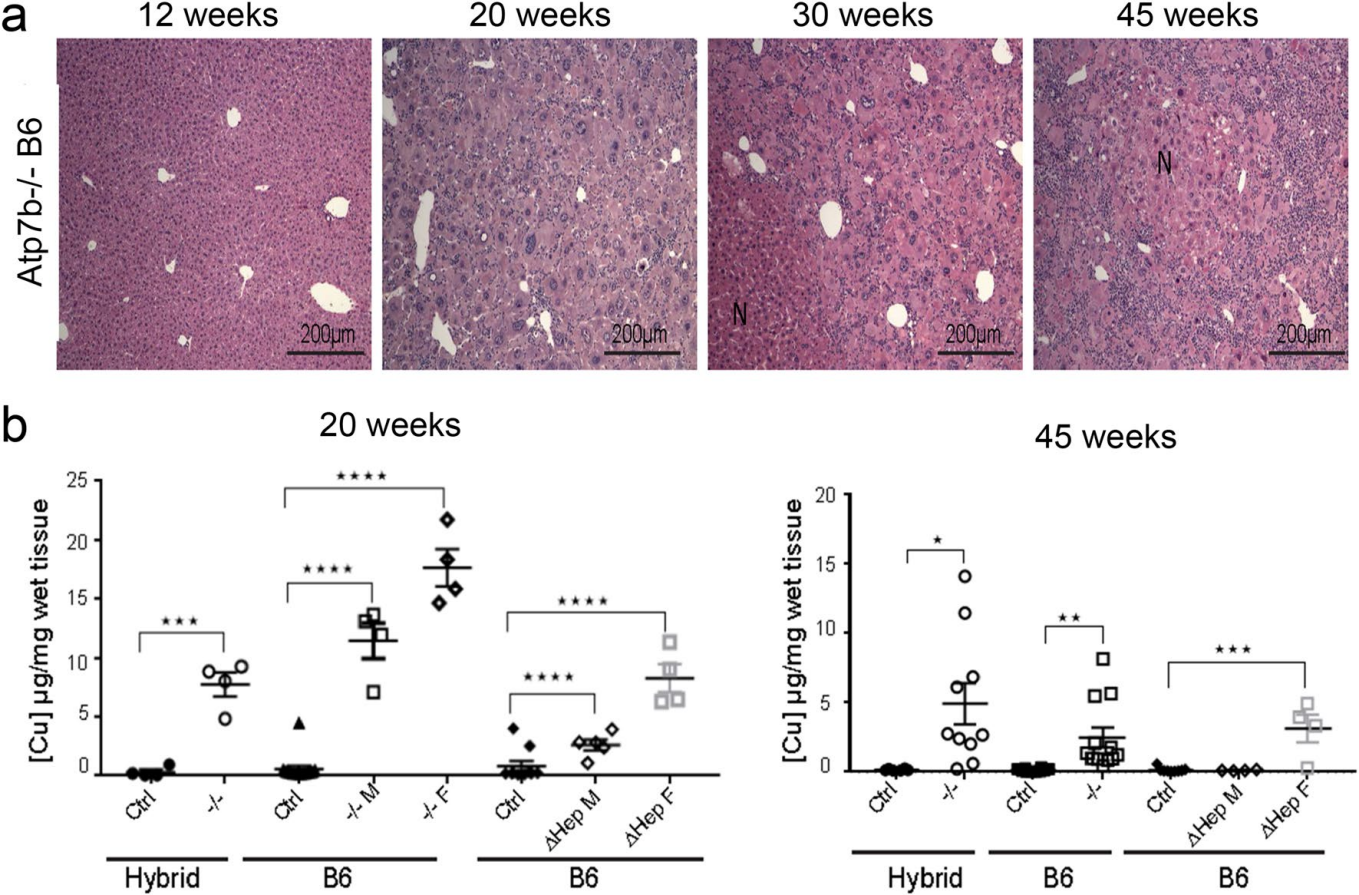
Time dependence of pathology development is not proportional to total Cu content of the liver tissue. (**a**) Representative H&E staining of *Atp7b*^*−*/*−*^-B6 liver sections at different ages. *Atp7b*^*−*/*−*^-B6 mice had extensive hepatocyte loss, degeneration, and mononuclear inflammation at 20 weeks that progressed further with age. At 12 weeks liver histology is essentially normal (**b**) Cu measurements in the livers of global (*−*/*−*) or hepatocyte-specific (ΔHep) Atp7b knockouts and corresponding controls (Ctrl- wildtype and heterozygous mice) on a Hybrid or B6 background at 20 weeks and 45 weeks. (n = 4–12). “F”-females; “M”-males. No sex differences in Cu values was observed for controls and these samples . The data were analyzed using one-way ANOVA. *p* values ≤ 0.05 was considered statistically significant. *p value ≤ 0.05, **p value ≤ 0.01, ***p value ≤ 0.001, and ****p value ≤ 0.0001.

To evaluate pathologic changes in *Atp7b*^*−*/*−*^ strains more carefully, we compared the timeline of pathology development in *Atp7b*^*−*/*−*^ livers. As previously reported, the *Atp7b*^*−*/*−*^-hybrid mice showed progressive hepatitis, hepatomegaly and karyomegaly at 12, 20, and 30 weeks ^18^. Similar pathology was observed in *Atp7b*^*−*/*−*^-B6 mice (Fig. 5a); however, changes in the gross liver morphology and histologic abnormalities in these animals developed at a much older age compared to *Atp7b*^*-/–*^hybrid mice. Specifically, in *Atp7b*^*-/–*^*B6* mice, liver histology appears normal at 12 weeks (Fig. 5a) in contrast to much more affected *Atp7b*^*-/–*^hybrid livers (Suppl. Fig. S2). In addition, only few *Atp7b*^*-/–*^*B6* animals showed nodules at 30 weeks, which are common for *Atp7b*^*-/–*^ hybrid mice at this age. Nevertheless, almost all *Atp7b*^*-/–*^*B6* animals had nodule formation at 45 weeks (Fig. 2). In other words, the genotype of the background strains influences the time of the disease onset, but the overall patterns of pathologic changes in *Atp7b*^*−*/*−*^ livers are similar.

### Hepatic Cu content does not predict the timing of disease onset

Differences in the amount of accumulated Cu may explain different timing of the pathology onset and phenotypic variability in the *Atp7b*-mutant strains. Consequently, we compared the liver Cu content in all mutant strains and the background-matched controls. The tissue samples were processed identically and measured in parallel. As expected, all three *Atp7b*-deficient strains showed elevated hepatic Cu when compared to the respective age- and background-matched controls (Fig. 5b). However, there was no correlation between a total hepatic Cu and liver histopathology. The *Atp7b*^*−*/*−*^ hybrid mice have the most severe liver injury and the *Atp7b*^*ΔHep*^*-B6* females have the mildest liver injury, yet these mice accumulate similar amounts of Cu (*Atp7b*^*∆Hep*^ males accumulated less Cu than *Atp7b*^*∆Hep*^ females). Both male and female *Atp7b*^*-/–*^*B6* animals had the greatest Cu accumulation, compared to other strains (Fig. 5b). *Atp7b*^*−*/*−*^ B6 females had twice as much Cu as *Atp7b*^*−*/*−*^ hybrid females, yet the *Atp7b*^*−*/*−*^ B6 animals had a significantly later onset of liver disease (see above). Clearly, factors additional to total hepatic Cu content influence the onset of liver injury.

### Changes in liver proteomes reflect the extent of liver disease

To provide a mechanistic framework for the observed phenotypic differences, we characterized the liver proteomes of mutant strains and respective controls at 20 weeks using Tandem Mass Tag (TMT) mass-spectrometry. This methodology enables accurate quantitative comparison of multiple samples because they are analyzed simultaneously, and proteins are identified in multiple samples within the same mass-spectrometry run. The age of 20 weeks was chosen for the analysis, because it best reflects the strain-specific differences, i.e. no disease in *Atp7b*^*ΔHep*^-B6 mice, a mild disease in *Atp7b*^*-/–*^B6 animals, and a fully developed pathology in *Atp7b*^*−*/*−*^-hybrid mice. Two datasets (10 animals per dataset) were generated with a combination of genotypes to decrease the batch effect and increase reproducibility, and the findings were verified using two additional datasets with different combinations of samples (see methods for details). Approximately 7500 proteins were identified for each condition; in each dataset, over 6000 common proteins were present in all samples (Table S2).

**Figure 6:**
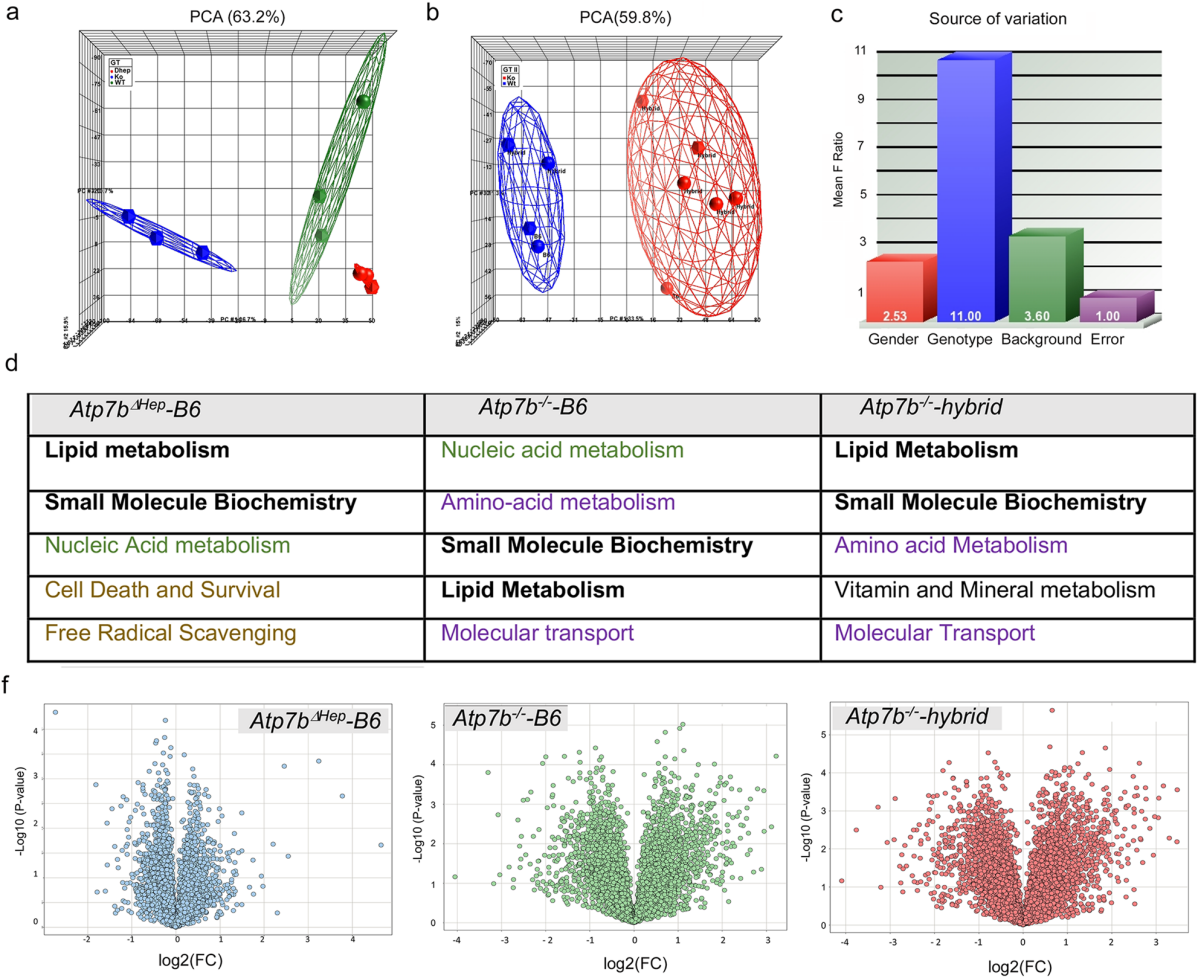
Changes in proteomes reflect the functional state of liver tissue. (**a**) The PCA plot illustrates complete segregation of WT-B6 (green), *Atp7b*^*ΔHep*^*-*B6 (red), and *Atp7b*^*-/–*^B6 (blue) protein profiles. (**b**) The PCA of protein profiles from *Atp7b*^*-/–*^hybrid and *Atp7b*^*-/–*^B6 livers and the respective backgrounds controls demonstrate differences between the WT (blue) proteomes and *Atp7b*^*−*/*−*^proteomes (red) that are independent of strain background. (**c**) Variance analysis identifies the Atp7b status (genotype) as the major contributor to changes in the liver proteomes along with gender and genetic background as minor contributors. (**d, top**) The most significantly affected cellular functions in each *Atp7b* mutant strains identified by the Ingenuity pathway analysis (https://digitalinsights.qiagen.com/products-overview/discovery-insights-portfolio/analysis-and-visualization/qiagen-ipa/). The cellular functions altered in all mutant strains are shown in bold; the processes affected in both global knockouts are in purple; pathways more strongly upregulated in the hepatocyte-specific knockout (compared to the background-matched global knockout) are in light brown. Nucleic acid metabolism (green) is strongly dis-regulated in both global and hepatocyte-specific knockouts when compared in the same mass-spectrometry run (**d, bottom**) The corresponding volcano plots depicting protein changes in each *Atp7b* mutant liver in comparison to the respective background matched control (The data were visualized using Spotfire DecisionSite with Functional Genomics v9.1.2).

Principal component analysis (PCA) revealed excellent segregation of the proteomes according to the *Atp7b* status (WT, KO, ΔHep) in mice with the same, B6, genetic background (Fig. 6a). There was also a co-segregation of the proteomes for global knockouts and a clear difference between the global *Atp7b*^*−*/*−*^ knockouts and the WT mice, independently of the strain genetic background (Fig. 6b).

Since the experimental samples included several variables (genetic background, *Atp7b* deletion, and gender), variance analysis was used to determine which of these parameters had the most significant impact on changes in the proteome (Fig. 6c). Inactivation of *Atp7b* was identified as the major factor affecting the proteome, and gender and genetic background were also found to contribute to proteome variability, in agreement with the functional data (see above). In other words, overall changes in proteomes reflect the phenotypic state of the liver.

### Hepatocytes’ response to Cu overload

To better understand how hepatocytes alone respond to Cu elevation, we analyzed changes in the proteome of *Atp7b*^*ΔHep*^*-B6* livers in comparison to WT-B6 livers. The fold-change values exceeding two standard deviations of the mean (|σ| > 2) were used as a cut-off, yielding a total of 1417 changed proteins (Suppl Table 2). Analysis of these proteins using Ingenuity showed significant associations of altered proteins with lipid homeostasis, small molecule biochemistry, nucleic acid metabolism, cell death and survival, and free radical scavenging (Fig. 6d). The most changed proteins (|σ| > 6; *p* < 0.01) protect hepatocytes against metal accumulation/redox misbalance or regulate basic metabolic functions. Specifically, metallothioneins MT1 and MT2 (the main endogenous Cu chelators) and nicotinamide nucleotide trans-hydrogenase (the enzyme involved in NADPH synthesis) were significantly elevated in *Atp7b*^*ΔHep*^*-B6* livers*,* whereas proteins involved in regulation of transcription/translation (Mrrf and Tcea3), protein stability (Fbxl 18), trafficking (Lrrc6), and signaling (Flcn, Gab1) were significantly down-regulated (Suppl. Table 3). Given the lack of abnormal histopathology in *Atp7b*^*ΔHep-*^*B6* mice, these data suggest that *Atp7b*^*ΔHep*^*-B6* animals are able to compensate for the Cu induced metabolic stress by increasing its redox and metal chelating capacity, while partially suppressing the energy consuming processes.

### Systemic Cu misbalance modulates the hepatocytes response to Cu overload

**Figure 7:**
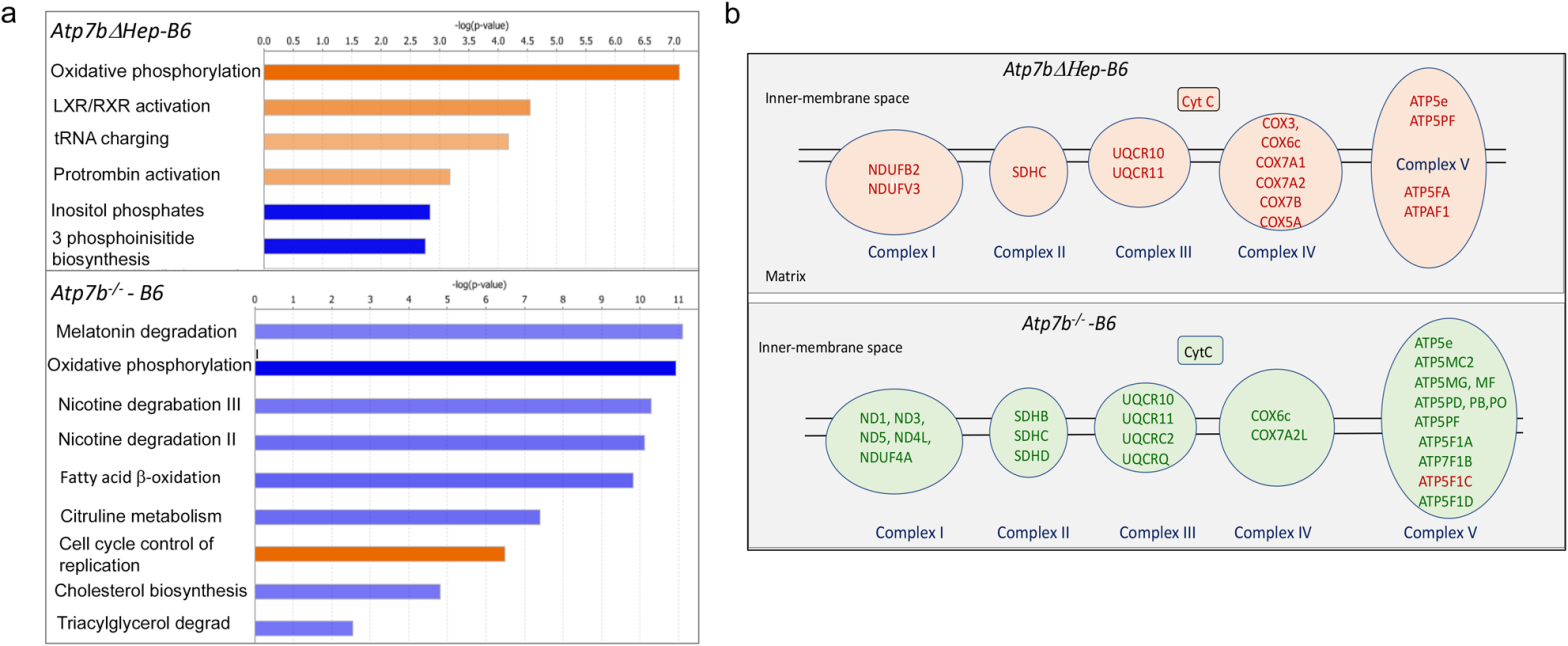
Comparison of the altered pathways in *Atp7b*^*ΔHep*^*-*B6 and *Atp7b*^*-/–*^B6 livers using Ingenuity Pathway Analysis (IPA). (**a**) The most significantly changed pathways (|Z| score > 2.5 for *Atp7b*^*-/–*^B6 animals and > 2.3 for *Atp7b*^*ΔHep*^*-*B6 pathways. The data were generated using samples analyzed within the same mass-spectrometry run; n = 3 per each genotype. Orange color indicates the upregulated pathways and blue color – down-regulated pathways. The complete list of affected pathways is given in Suppl Table 4 and Suppl Table 5. (**b**) Components of the respiratory chain in mitochondria that are significantly changed in response to Cu accumulation in *Atp7b*^*ΔHep*^*-*B6 and *Atp7b*^*-/–*^B6 mice. Upregulated proteins are labeled in red, down-regulated proteins are in green. Data generated and visualized using IPA (https://digitalinsights.qiagen.com/products-overview/discovery-insights-portfolio/analysis-and-visualization/qiagen-ipa/).

The phenotypic comparison of *Atp7b*^*ΔHep*^*-B6* and *Atp7b*^*-/–*^*B6* (above) suggested that the global deletion of Atp7b modifies the liver response to Cu overload. Consistent with the phenotypic differences, there were striking differences between the liver proteomes of these animals. When compared to WT-B6 liver, both the number of altered proteins and the fold-changes were larger in *Atp7b*^*-/–*^*B6* livers than in *Atp7b*^*ΔHep*^*-B6* livers (Fig. 6f). Also, more pathways were altered in *Atp7b*^*-/–*^*B6* livers compared to *Atp7b*^*ΔHep*^*-B6* livers (Suppl. Table 4 and Suppl. Table 5). In addition, changes in *Atp7b*^*-/–*^*B6* livers reflected mostly metabolic inhibition, whereas *Atp7b*^*ΔHep*^*-B6* livers showed many upregulated pathways (Fig. 7a). The most unexpected observation was the opposite change in the abundance of respiratory chain components, which were increased in *Atp7b*^*ΔHep*^*-B6* livers and decreased in *Atp7b*^*-/–*^*B6* livers (Fig. 7b). Opposite changes were also observed for LPS/IL1-mediated inhibition of RXR function, nicotine degradation, cell cycle control of chromosome remodeling, and melatonin degradation.

In agreement with the phenotypic differences (low serum glucose and glycogen deposition), the protein machinery involved in glucose uptake, utilization, and storage was downregulated in *Atp7b*^*-/–*^*B6* mice compared to the respective WT-B6 mice. Specifically, the levels of glucose transporters SLC2A2, SLC2A9, and SLC37, glucokinase, the catalytic subunit of glucose-6-phosphatase, and glycogen synthase 2 were significantly decreased. By contrast, in *Atp7b*^*ΔHep*^*-B6* animals, only glucokinase levels were slightly lower (1.27-fold compared to a 2.1-fold decrease in *Atp7b*^*-/–*^B6 mice and 3.5-fold decrease in the *Atp7b*^*−*/*−*^ hybrid strain).

### Global inactivation of Atp7b impact similar cellular pathways in Atp7b^-/–^B6 and Atp7b^-/–^hybrid livers

**Figure 8:**
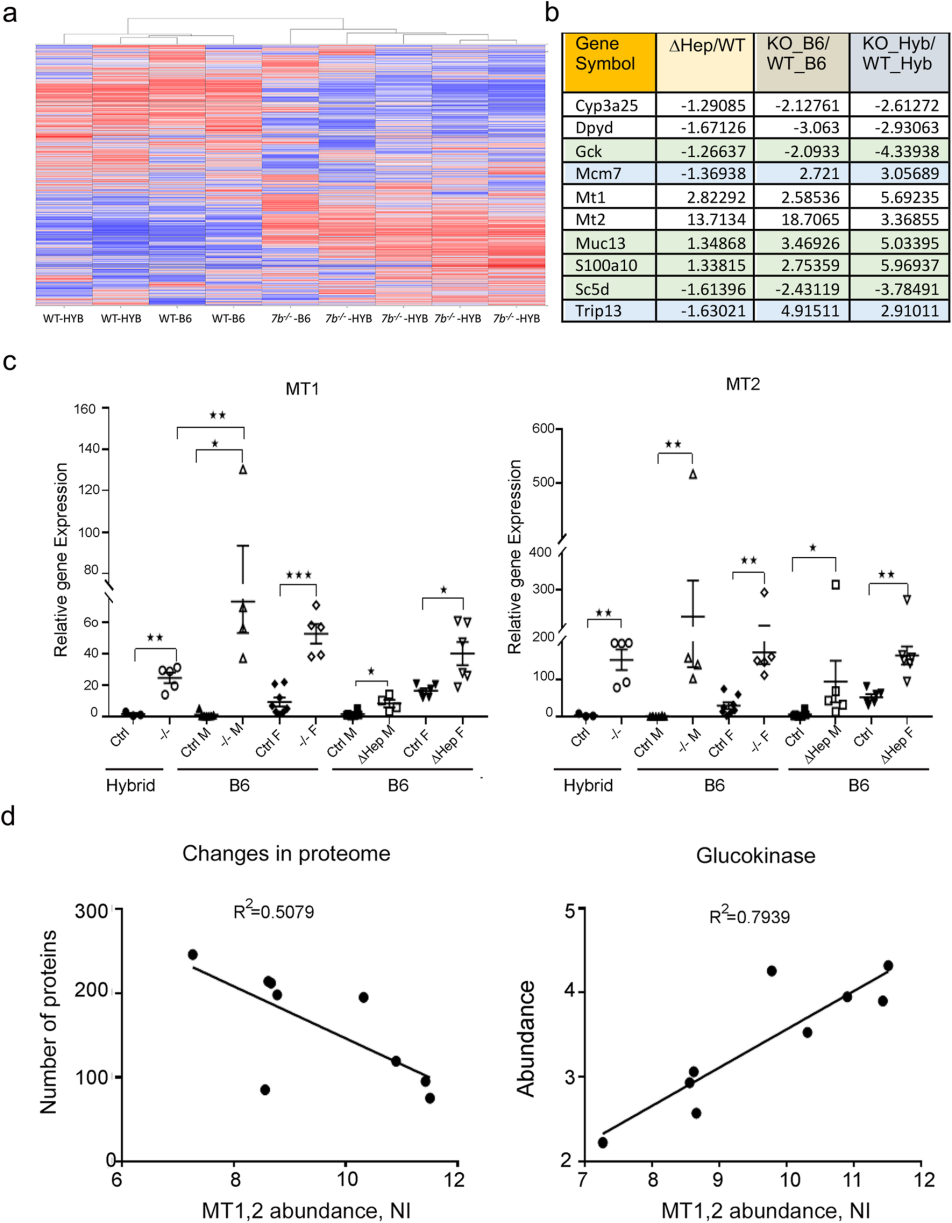
Comparison of Atp7b mutant mice suggests potential markers and modifying factors. (**a**) Unsupervised clustering of protein profiles from the dataset # 2 (see methods) was done using Spotfire DecisionSite with Functional Genomics v9.1.2 (https://edelivery.tibco.com/storefront/eval/tibco-spotfire-decisionsite-for-functional-genomics/prod10196.html) and presented as a heat map; the map illustrates marked differences between the wt animals and *Atp7b*^*−*/*−*^ global knockouts and presence of similarly changed proteins in *Atp7b*^*−*/*−*^ mice on different genetic backgrounds heat map (**b**) The most significantly dysregulated proteins (|S|= 6, *p* value < 0.05) detected in all *Atp7b*-mutant mice. Green color highlights protein with changes in abundance that reflect the extent of liver pathology; blue indicate proteins that are oppositely changed in the hepatocyte-specific and global knockouts; (**c**) Upregulation of metallothioneins MT1 and MT2 in *Atp7b*-deficient strains. One-way ANOVA and a two-tail t-test for individual pairs (shown) were used (*p value ≤ 0.05, **p value ≤ 0.01, ***p value ≤ 0.001) and yielded same conclusions. “Ctrl” indicates either *Atp7b*^+*/*+^ or *Atp7b* ^+*/-*^ animals; “M”—males; “F”—females. (**d**) *Left:* Correlation between the abundance of metallothioneins MT1 and MT2 in individual *Atp7b*^*−*/*−*^ animals and the number of proteins in their liver proteome changed more than1.5-fold compared to the averaged wild-type on the same genetic background. Right: Correlation between abundances of MT1 and MT2 and levels of glucokinase in individual *Atp7b*^*−*/*−*^ animals. “NI” indicates a normalized protein intensity.

The phenotypic data (weight measurements, liver morphology and function, metabolic parameters) and the PCA of protein profiles suggested that the overall molecular changes in two global *Atp7b*^*−*/*−*^ knockouts are similar despite different timing of disease onset. We tested this hypothesis using unsupervised clustering of protein profiles (Fig. 8a and Suppl. Table 6), and also compared changes in the proteomes using volcano plots and Ingenuity pathway analysis (Fig. 6d,f).

These experiments revealed an overall similar liver response to Cu overload in global *Atp7b* knockouts. Both *Atp7b*^*-/–*^*B6* and *Atp7b*^*-/–*^*hybrid* livers show changes in the abundance of a large number of proteins (Fig. 6f), along with a common enrichment in the amino acid and lipid metabolism, small molecule biochemistry, and molecular transport (Fig. 6d, Suppl. Table 4 and Suppl. Table 7). Oxidative phosphorylation, fatty acids and steroid metabolism, nicotine and melatonin degradation are inhibited in both global knockouts, whereas IL6 signaling and IL1-mediated inhibition of RXR function are enhanced (Suppl. Table 4 and Suppl. Table 6). The RhoGDI signaling is diminished in both strains. Dysregulation of RXR-paired nuclear receptors (FXR, LXR, RAR, and aryl hydrocarbon receptor) is observed in both global knockouts in agreement with the previously published data^10–12^. (For complete lists of altered pathways, see Suppl Tables 4, 5, 7).

We also identified proteins that were reproducibly and significantly changed in all Atp7b mutant livers (Fig. 8b). In particular, glucokinase (Gck) and lathosterol oxidase (Sc5d) were consistently down-regulated in all three strains, whereas mucin Muc13 and a calcium binding protein S100A10 were significantly upregulated. Moreover, the extent of these protein dysregulation paralleled the extent of liver pathology, suggesting that these proteins could be markers of WD onset and progression.

### Factors modifying WD onset in mice

Although gross pathologic changes and overall changes in protein profiles of *Atp7b*^*−*/*−*^ hybrid and *Atp7b*^*-/–*^B6 livers were similar (Figs. 2a, 8a), the timing of disease onset in these strains differed significantly, pointing to strain-specific liver responses. Consequently, we searched for the strain-related differences that may explain an earlier disease onset in *Atp7b*^*-/–*^hybrid mice. Metallothioneins (MT1 and MT2) are endogenous, metal chelating proteins; they tightly bind Cu and limit Cu availability. Each MT can bind 10–11 atoms of Cu per protein^24,25^, and therefore even a 10% difference in MT levels between the strains could translate into a marked difference in their Cu sequestration capacity. We hypothesized that the “non-sequestered” Cu, rather than total Cu, was important for pathology development and that differences in MTs abundance may explain discrepancies between the total liver Cu and the extent of pathology.

MT1 and MT2 proteins were highly upregulated in both *Atp7b*^*−*/*−*^ strains, but the magnitude of upregulation differed significantly between the strains. *Atp7b*^*−*/*−*^ hybrid animals had a 12-fold and 11-fold increase in MT1 and MT2 protein levels, respectively, compared to the age-matched WT-hybrid animals. In contrast, *Atp7b*^*-/–*^*B6* mice showed a 50-fold increase for MT1 and a 30-fold increase for MT2 compared to WT-B6 controls. Within the same mass-spectrometry experiment, when the protein signal intensities can be directly compared, not only the relative fold change but the actual abundances of MT1 and MT2 were higher in *Atp7b*^*-/–*^*B6* mice compared to *Atp7b*^*−*/*−*^ hybrid mice (Suppl. Table 2).

Hepatic mRNA levels of MT1 and MT2 are sensitive to Cu levels and were previously shown to be elevated in animal models of WD^26–28^. Consequently, to verify our results, we used the mRNA levels as a proxy for the abundance of MT proteins. QPCR analysis confirmed both the upregulation and higher abundance of metallothioneins in *Atp7b*^*-/–*^B6 livers compared to *Atp7b*^*−*/*−*^-hybrids—for both males and females (Fig. 8c). The median increase in MT1 transcripts in *Atp7b*^*−*/*−*^-hybrid livers was 50-fold, whereas *Atp7b*^*-/–*^B6 showed a ~ 100–150-fold increase when compared to the respective WT controls. For MT2, the upregulation was higher in B6-males than in B6-females, and the total level of MT2 transcripts was similar in *Atp7b*^*-/–*^B6 and *Atp7b*^*−*/*−*^-hybrid liver samples.

Taken together, the transcriptome and proteome data show that *Atp7b*^*-/–*^B6 livers produce significantly more MT1/2 and therefore have a higher Cu sequestering capacity compared to *Atp7b*^*−*/*−*^-hybrid livers. We hypothesized that the extent of pathological changes could be determined by the liver “Cu-sequestration capacity” rather than total Cu. To test this hypothesis, we correlated the overall liver response (the number of significantly changed proteins: 1.5 fold, *p* value < 0.05) in individual animals with the metallothionein abundance in their livers, and observed inverse correlation (Fig. 8d left panel), i.e. higher metallothionein abundance is associated with fewer significantly altered proteins. We then examined whether the abundance of potential protein “markers” (see above) correlates with MT1/2 abundance in individual animals. The most significant correlation was seen for Gck (glucokinase) and the (MT1 + MT2) levels. The larger decrease in Gck abundance correlated with lower levels of metallothioneins (i.e. lower Cu sequestration capacity), whereas more Gck was present in animals with higher levels of metallothioneins (Fig. 8d).

## Discussion

In this study, we show that the overall pattern of pathological changes in animals with global inactivation of Atp7b is similar at the molecular and cellular levels in strains with different genetic background. We identified the cellular pathways affected in all Atp7b-deficient animals, highlighted a significant impact of systemic Atp7b inactivation on liver response to Cu overload, and reconciled some discrepancies that existed in the literature.

Previous characterization of animal models for WD uncovered important aspects of disease pathogenesis, including involvement of mitochondria^15,16,29^, autophagy^30^, the role of nuclear receptors^10,11^, along with a significant impact of Cu overload on Zn balance^31^. These studies stimulated search for new therapeutic modalities, including small molecule drugs, cell replacement therapies, and gene therapies^10,29,32,33^. Hepatocyte-directed *ATP7B* gene transfer has recently shown significant promise in improving liver morphology and function in *Atp7b*^*−*/*−*^ mouse models of WD^33–35^. In future studies, it would be important to show whether the Atp7b deficit is corrected in hepatocytes alone or also in other non-parenchymal cells when the liver function is restored.

Our current results on *Atp7b*^*ΔHep*^-B6 mice along with the previous work illustrate that the unremarkable liver histology, normal serum transaminases, and the lack of copper in the urine are insufficient indicators of healthy liver metabolism – they only indicate that liver is able to cope with the existing metabolic insults.

Therefore, the observed incomplete penetrance of human WD could be a manifestation of subclinical changes and be explained, in part, by insufficiently sensitive methodologies employed for characterization of patients’ liver status. Analysis of more sensitive markers of liver injury such as presence of characteristic protein and/or metabolic markers (see below) may yield a more complete understanding of liver status/vulnerability in individuals with mutations in ATP7B.

Although *Atp7b*^*ΔHep*^-B6 mice do not show any gross hepatic abnormalities, many pathways affected in the obviously diseased *Atp7b*^*−*/*−*^ knockouts are also compromised in *Atp7b*^*ΔHep*^animals, predisposing them to obesity and liver steatosis. Taken together, our data indicate that when Atp7b inactivation is limited to hepatocytes, liver upregulates proteins involved in vulnerable pathways (redox balance, mitochondria function) to compensate for functional deficiencies caused by Cu overload. This compensatory capacity is lost under conditions of systemic Atp7b inactivation.

Some of our findings were surprising and therefore informative. First, we found that Cu accumulation in hepatocytes had opposite effect on the mitochondria proteins in the global *Atp7b*^*-/–*^*B6* knockouts and *Atp7b*^*ΔHep*^*-B6* animals. Dysregulation of mitochondria function in WD was previously reported in humans and animals^15,16,29^, and the suggestion was made that Cu causes a destruction of mitoproteome^16,29^. In our study, the differentially expressed mitochondrial proteins were all encoded in the nucleus and not in the mitochondria, which argues against Cu directly impacting stability of mitochondria proteins or DNA. This conclusion is in agreement with a previous report showing no hepatic mtDNA depletion in livers of toxic milk mice (another animal model for WD^15^). Our proteomics results and the previous work measuring effects of Cu overload on the mitochondria redox balance^36^ offer a more nuanced mechanism. Specifically, our data suggest that Cu accumulation triggers changes in redox balance, which is compensated by upregulation of enzymes involved in NADPH and glutathione synthesis. In global Atp7b knockouts, the loss of building blocks necessary for NADPH and glutathione synthesis (nucleotides and amino acids- see altered pathways) compromises this protective compensatory response causing the onset of liver disease.

Secondly, we found that the LXR/RXR and FXR/RXR-dependent pathways show different changes in the global knockouts and *Atp7b*^*ΔHep*^ animals. This finding argues against direct inhibition of LXR and/or FXR function by Cu binding, although further measurements of Cu effects on LXR and FXR in vivo are necessary to firmly establish the mechanism. Interestingly, we observed strong upregulation of estrogen sulfotransferase (Sult1e1) in the livers of Atp7b global knockouts on both genetic backgrounds but not in *Atp7b*^*ΔHep*^ livers. Sult1e1 modifies the endogenous LXR agonist, 24-hydroxycholesterol, leading to LXR inhibition^37^. Sult1e1 is also a transcriptional target of LXR^37^. Thus, the activity of nuclear receptors in *Atp7b*^*−*/*−*^ liver may reflect a complex interplay of metabolites generated by the Cu-altered enzymes as well as transcriptional activities of LXR and FXR proteins per se. Studies with the known LXR and FXR agonists would help not only to better describe the role of the LXR and FXR-dependent pathways in WD onset and progression, but potentially develop new therapies targeting these pathways.

Proteins involved in lipid metabolism were altered in all three Atp7b mutant strains, but were differentially dysregulated. The abundance of long- and very long-chain acyl-CoA synthase/transporter Slc27a2 and Fabp5 (a cytosolic carrier for long-chain fatty acids) was decreased in both global knockouts more than twofold (*p* = 0.0002–0.0013) but not in *Atp7b*^*ΔHep*^*-B6* mice. Instead, the *Atp7b*^*ΔHep*^*-B6* mice upregulate Fabp7, which is not changed in global knockouts. It is tempting to speculate that more pronounced deficits of lipid metabolism in global knockouts compared to *Atp7b*^*ΔHep*^*-B6* mice may originate in the intestine, where we previously found a significant impact of Atp7b inactivation on processing of dietary fat^22^.

WD presents many challenges for diagnosis and treatment. Although the etiology of WD has been long established, clear markers that differentiate WD from other liver disorders are yet to emerge. Recent studies of metabolites in the serum of WD patients suggest existence of the WD “signature”^38^, and our studies further support this concept. Despite significant variation in the time of disease onset, *Atp7b*^*−*/*−*^ animals on different genetic backgrounds consistently show changes in lipid metabolism, glucose utilization, pyrimidine biosynthesis, and amino acid metabolism. Furthermore, analysis of the most significantly changed proteins illustrates that not only the abundance of these proteins is altered in all Atp7b-mutant strains characterized in this study, but the magnitude of changes is proportional to the extent of pathologic changes in the liver. It would be interesting to see whether the decreases in glucokinase and lathosterol oxidase along with the elevation of mucin-13 and S100a10 protein occur in human WD.

It has been recently suggested that changes in mouse liver secretome do not recapitulate changes in human serum^39^. This conclusion could be premature. The discrepancy between the human and mouse secretome observed in^39^ could be caused by the fact that the chelator-treated patients were compared to untreated mice and a small number of proteins was analyzed due to limited access to human material. The comparison of proteomic profiles generated in our study with the metabolic and transcriptomic changes reported by Sarode et al.^38^, Burkhead et al.^40,41^ and Wooton-Kee et al.^12^ show significant similarities between humans and animals in their liver response to Cu overload. Changes of glucose metabolism appear in all strains, just at different ages, and are also seen in humans. Sarode et al. identified the WD-related metabolic markers that reflect changes in nitrogen metabolism, glutamate/glutamine metabolism, the Val/Leu/Ile biosynthesis and degradation, and glutathione metabolism (25).These findings are in full agreement with the dysregulation of metabolism of glutamine family amino acids (caused by significant decreases in abundance of ARG1, GLS2, GLUL and IDH1), inhibition of urea cycle, changes in Val, Leu, and Ile degradation and upregulation of glutathione synthesis predicted by the proteomics studies of our *Atp7b*^*−*/*−*^ mice (see Suppl. Tables). Focus on these pathways, commonly changed in WD patients and Atp7b-deficient animals, could be useful when testing the efficacy of new drugs and treatments in preclinical studies.

It is significant that changes in some proteins are detected before pathologic changes in the liver. Therefore, unique metabolic markers in combination with distinct proteomic profiles may help to diagnose patients earlier and provide a more accurate monitoring throughout the course of the disease. Further studies using human samples are necessary to identify the WD-specific protein-based signatures in the serum and/or liver. The results of our study could be used as an initial guide to search for the markers when the amount of human material is insufficient for unbiased omics assays.

Gender differences are well documented in human WD. Similarly, significant gender-related differences in Cu levels, metabolic changes, and histopathology were observed for our mouse models of WD emphasizing the importance of including both sexes in testing new therapeutic approaches.

Finally, our data show that the protein levels of metallothioneins differ significantly not only among the *Atp7b*^*−*/*−*^ animals from different strains, but also between the individual animals with the same genetic background. This observation suggests that natural differences in MT1/2 levels may result in different capacity to buffer accumulated Cu and thus modulate the liver response. Metallothioneins abundance is regulated not only by the tissue metal content (zinc and copper), but also by the cellular redox status, serum growth factors, and by the ligands of FXR nuclear receptor^42^. Natural variability in these regulatory factors may further attenuate metallothionein abundance and hence Cu sequestration capacity of tissues. Going forward, it would be interesting to determine whether levels of metallothioneins in humans can be used as predictors of the timing of disease onset and pathology development.

## Supplementary Information

Supplementary Information 1.

Supplementary Information 2.

Supplementary Information 3.

Supplementary Information 4.

Supplementary Information 5.

Supplementary Information 6.
