# Supplementary material for: Systemic deletion of Atp7b modifies the hepatocytes’ response to copper overload in the mouse models of Wilson disease
Source: Sci Rep. 2021 Mar 11;11:5659. doi: 10.1038/s41598-021-84894-3 (PMC7952580; doi:10.1038/s41598-021-84894-3)
Supplement: Supplementary file 1 — Supplementary Information 1. [file 41598_2021_84894_MOESM1_ESM.pdf]

SUPPLEMENT

SYSTEMIC DELETION OF ATP7B MODIFIES THE HEPATOCYTES' RESPONSE TO COPPER OVERLOAD IN THE MOUSE MODELS OF WILSON DISEASE

Abigael Muchenditsi[1], C. Conover *Talbot Jr*.[2], Aline Gottlieb[1], Haojun Yang[1], Byunghak Kang[3]*, Tatiana Boronina[4], Robert Cole[4], Li Wang[1], Som Dev[1], James P. Hamilton[5], and Svetlana Lutsenko[1*]

Suppl. Table 1. Sequencing of primers used for genotyping and qPCR

**Primers for qPCR**	
Gapdh	5'-AACTTTGGCATTGTGGAAGG-3'<br>5'-CACATTGGGGGTAGGAACAC-3'
MT1	5’-CACTTGCACCAGCTCCTG-3’<br>5’-GAAGACGCTGGGTTGGTC-3’
MT2	5’-GCAAACAATGCAAATGTACTTCC-3’<br>5’-CTATTTACACAGATGTGGGGACC-3'
**Primers for genotyping**	
Neo Del	5'- CGTCATAGCAAAGCTTGGTAACC -3'<br>5'- CTTAAGTGCTGGATATGGGCATG-3'
Cre	5'-AATGCTTCTGTCCGTTTGCCGGT-3'<br>5'- CCAGGCTAAGTGCCTTCTCTACA-3'
Atp7b	FWD 5’-GGCATTGTGAACATCAAGGTG-3’<br>REV WT 5’- ATGGCTGTCTGCAGGAACA-3’<br>REV KO 5’-TCGAGATCCACTAGTTCTAGC-3'

Note: Neo Del and Cre primers were used to detect the presence of Lox P sites and Cre, respectively. Atp7b primers were used for detection of the full length Atp7b (Rev Wt) or Atp7b deletion (Rev KO)

Suppl. Table 2

Individual abundances, fold changes, and p-values for all identified proteins.

Suppl. Table 3 The most significantly changed proteins in *Atp7b*$^{\Delta Hep}$-B6 Livers

Gene Symbol	*Dhep_1Way vs. B6_WT Lin(FC)*	*Dhep_1Way vs. B6_WT Log2(FC)*	*SD Binned Dhep vs B6_WT Log2(FC)*	*Dhep_1Way vs. B6_WT (p-val)*	*Protein name*
Nnt	9.45321	3.2408	> +6s	0.000443191	nicotinamide nucleotide transhydrogenase
Hyi	5.49303	2.4576	> +6s	0.000561652	hydroxypyruvate isomerase (putative)
Mt2	13.7134	3.77751	> +6s	0.00225461	metallothionein 2
Mt1	2.82292	1.49719	+6s	0.00491404	metallothionein 1
Ppp3r1	2.0668	1.0474	+6s	0.00567732	Calcineurin subunit B type 1
Lrrc6	-3.50551	-1.80962	-6s	0.00131876	leucine rich repeat containing 6
Tcea3	-2.28529	-1.19238	-6s	0.00231609	transcription elongation factor A3
Mrrf	-2.16571	-1.11484	-6s	0.00265801	Ribosome-recycling factor, mitochondrial
Flcn	-2.37244	-1.24637	-6s	0.00356135	folliculin
Plscr3	-2.72431	-1.44589	-6s	0.00404638	phospholipid scramblase 3 (mitochondria)
Xiap	-2.03244	-1.02321	-6s	0.00603917	X-linked inhibitor of apoptosis protein
Fbxl18	-2.08415	-1.05946	-6s	0.00752284	F-box and leucine rich repeat protein 18
Gab1	-2.94225	-1.55692	-6s	0.00789806	GRB2-associated-binding protein 1
Pigu	-6.63674	-2.73047	= -6s	4.58E-05	Phosphatidylinositol glycan anchor biosynthesis class U protein

Suppl. Table 4

The canonical metabolic and signaling pathways significantly altered in Atp7b$^{-/-}$-B6 livers and arranged based on z-score values

Suppl. Table 5

The canonical metabolic and signaling pathways significantly altered in Atp7b$^{\Delta Hep}$ -B6 livers and arranged based on z-score values

Suppl. Table 6

The canonical metabolic and signaling pathways significantly altered in Atp7b$^{\Delta Hep}$ -hybrid livers and arranged based on z-score values

Suppl. Table 7

The least of genes used to generate a heat map shown in Fig.8a

Suppl. Fig. 1

B6-background

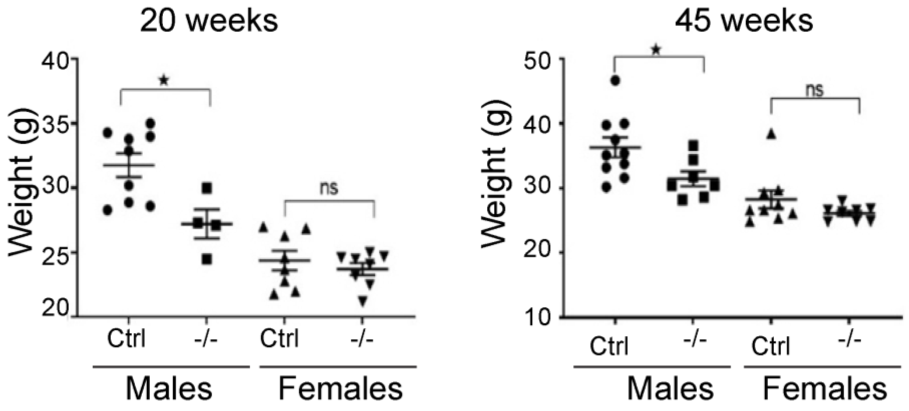

Hybrid-background

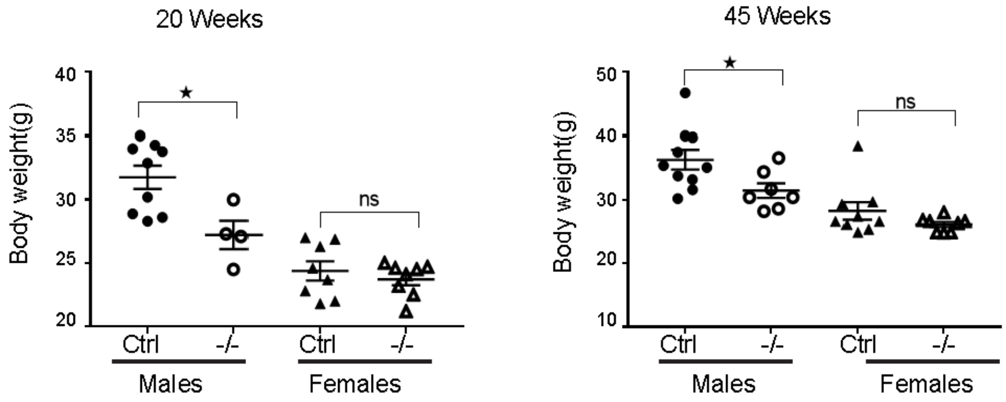

Fig S1. Weight measurements for *Atp7b*$^{-/-}$B6 and *Atp7b*$^{-/-}$hybrid global knockouts (-/-) and the respective age- and sex-matched wild-type controls (Ctrl) at 20 and 45 weeks. Male animals show significantly decreased body weight at both ages, whereas female animals were similar to controls. The data were analyzed using unpaired two-tailed T test. $^{*}P \leq 0.05$ is considered statistically significant and $P > 0.05$ not statistically significant, (ns)

Suppl. Fig.2

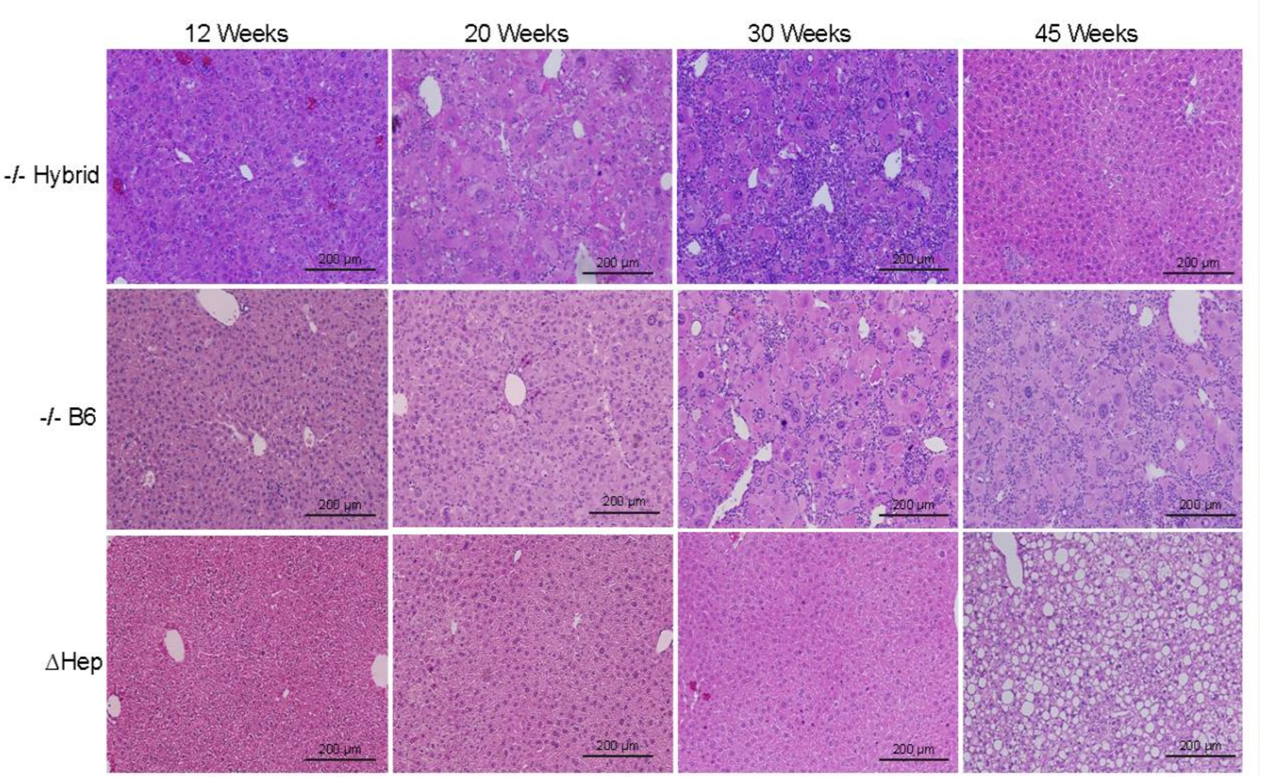

Fig S2. Time dependence of pathology development in different Atp7b mutant strains. ***Atp7b**$^{-/-}$**-hybrid,*** ***Atp7b**$^{-/-}$**-B6 and*** ***Atp7b**$^{\Delta Hep}$**-B6 (ΔHep)*** Representative images of paraffin embedded liver tissue sections (10 µm) stained with H&E at 12, 20, 30 and 45 weeks. The images were collected at 10x magnification using Olympus color station. The grey scale bar is 200 µm – the same in all images

Suppl. Fig. 3

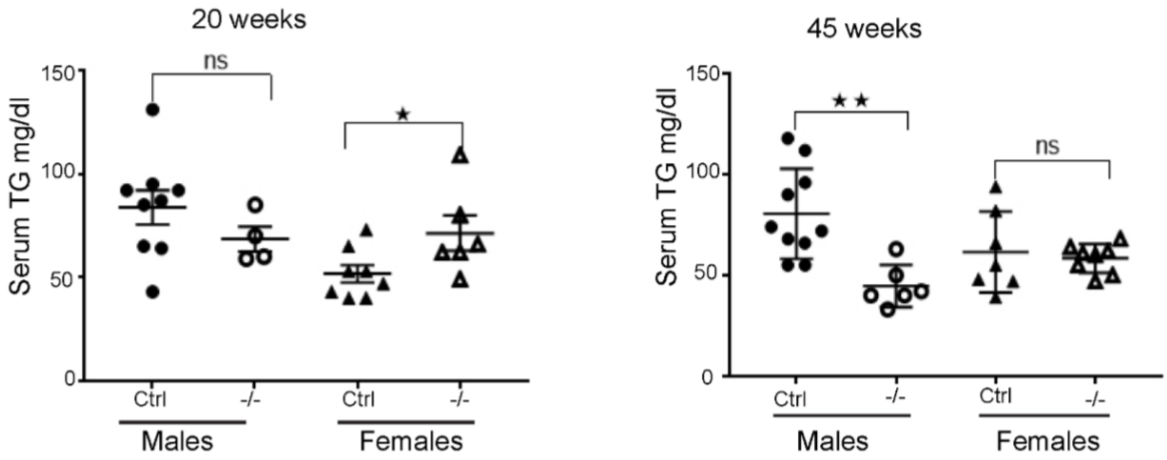

Fig S3. Serum Triglyceride measurements in Atp7b-/- B6 animals at 20 and 45 weeks. Blood was collected from animals during sacrifice and measurements done as mentioned in materials and methods. The data were analyzed using unpaired two-tailed t-test. $P \leq 0.05$ is considered statistically significant and $P > 0.05$ not statistically significant, (ns).
